# DNA Methylation by Bisulfite Next-Generation Sequencing for MLH1 and MGMT in Oral Squamous Cell Carcinomas and Potentially Malignant Disorders: An Integrative Analysis towards Field Cancerization

**DOI:** 10.3390/medicina58070878

**Published:** 2022-06-30

**Authors:** Elena Padin-Iruegas, Cintia M. Chamorro-Petronacci, Iria Sines-Cajade, Alejandro I. Lorenzo-Pouso, Andrés Blanco-Carrión, Alba Pérez-Jardón, Pilar Gándara-Vila, Mario Pérez-Sayans

**Affiliations:** 1 Human Anatomy and Embryology Area, Department of Functional Biology and Health Sciences, Faculty of Physiotherapy, University of Vigo, 36001 Pontevedra, Spain; mepadin@uvigo.es; 2 Health Research Institute of Santiago de Compostela (IDIS), Department of Oral Medicine, University of Santiago de Compostela (ORALRES Group), 15782 Santiago de Compostela, Spain; cintia.chamorro.petronacci@gmail.com (C.M.C.-P.); andres.blanco@usc.es (A.B.-C.); alba.perez.gonzalez@sergas.es (A.P.-J.); pilar.gandara@usc.es (P.G.-V.); perezsayans@gmail.com (M.P.-S.); 3 Department of Oral Medicine, University of Santiago de Compostela (MedOralRes Group), 15782 Santiago de Compostela, Spain; iria.sines@rai.usc.es

**Keywords:** DNA methylation, oral squamous cell carcinoma, next-generation sequencing, potentially malignant disorders, MLH1, MGMT

## Abstract

*Background and Objectives*: MGMT methylation is a well-described biomarker in several solid tumors and MLH1 seems to occur in the initial stages of oral carcinogenesis. The aims of this study were to evaluate MHL1 and MGMT methylation levels in oral squamous cell carcinoma (OSCC) and oral potentially malignant disorders (OPMDs), and to integrate this information with The Cancer Genome Atlas (TCGA) database. *Materials and Methods*: To determine the percentage of gene methylation in MLH1 and MGMT, pyrosequencing analysis was conducted. Samples were divided as follows: (1) patients diagnosed with OSCC (*N* = 16); (2) patients with OPDM who developed OSCC in the same location (*N* = 47); and (3) patients with OPDM who developed OSCC in a different location (*N* = 22). As a validation cohort in this study, data from The Cancer Genomic Atlas (TCGA) database, particularly regarding Head and Neck Squamous Cell Carcinoma, was used. *Results*: Overall MLH1 methylation levels of 8.6 ± 11.5% and 8.1 ± 9.2% for MGMT were obtained. With regard to MHL1, the OSCC presented the highest degree of methylation with 9.3 ± 7.3% (95%CI 5.1–13.6), and with regards to MGMT, the simultaneous malignancy group presented the highest degree of methylation with 10 ± 13.5% (95%CI 6–10), although no significant differences were found between the groups (*p* = 0.934 and *p* = 0.515, respectively). The estimated survival was higher for MGMT methylated cases (19.1 months, 95%CI 19.1–19.1) than for unmethylated cases (9.4 months, 95%CI 6–12.8), but not statistically significant. *Conclusions*: Our results did not show a correlation between MGMT and MLH1 methylation and any clinicopathological feature or survival in our institutional cohort. MLH1 methylation was present mainly in OSCC, whilst MGMT in OPMD represented a modest contribution to field cancerization, with an overall consistency with the TCGA database.

## 1. Introduction

Oral squamous cell carcinoma (OSCC) represents 95% of all cancers that can be found within the oral cavity [1]. It is considered neoplasia with a poor survival rate of around 50–60% [2,3]. OSCC etiology is highly debatable, although a certain genetic disposition coupled with other risk factors such as smoking and alcohol consumption, viral infections, actinic radiation and the malignant transformation of oral potentially malignant disorders (OPMDs) such as leukoplakia, erythroplakia, lichen planus, actinic cheilitis andr oral submucous fibrosis, are considered its main cornerstones [4,5,6,7,8].

MMR (mismatch repair) is one of the main DNA repair systems that relates to the homologous MutLS bacterial system (human MutS and MutL proteins) [9]. MLH1 (mutL homolog 1) is a human gene that plays a key role in the DNA duplication error reparation process, and likewise, it also plays a pivotal role in preserving genomic stability. On the other hand, methylguanine-DNA methyltransferase (MGMT) is a specific DNA damage repair protein which plays a key role in maintaining normal cell physiology and genomic stability [10]. Methylation of this promoter is a key predictor of whether alkylating agents can effectively control tumor cell progression [11].

The accumulation of genetic alterations may trigger the development of potentially malignant lesions and subsequently, carcinomas [12]. On the one hand, genetic alterations are considered irreversible changes to the DNA sequence, which result in oncogene activation or the deactivation of the tumor suppressor genes [13]. On the other hand, epigenetic alterations result in reversible and heritable changes. Nonetheless, these do not generate any DNA sequence alteration [10]. DNA methylation is an epigenetic mechanism in which a methyl group is transferred to the cytosine C5 position to form 5-methylcytosine [9]. This phenomenon regulates gene expression by recruiting the proteins that participate in gene repression, or by inhibiting the union of DNA transcription factors. DNA methylation usually takes place in the cytosines that precede a guanine, or alternatively, it can take place in CpG sites. This epigenetic modification is essential to silence the retroviral element and regulate the gene expression for specific tissues, and it is also essential for genomic imprinting and X chromosome inactivation [11].

MHL1 is one of the DNA MMR genes commonly found in Lynch syndrome and associated with germline mutations. Patients with MLH1 gene mutations usually develop colorectal cancers at an earlier age [14]. Different studies have shown that in most cases, MLH1 methylation occurs in the initial stages of oral carcinogenesis, although it can also appear, to a lesser degree, at more advanced stages. Therefore, it is worth noting that MHL1 methylation is an early process that continues throughout the tumor progression [15,16]. On the other hand, it has been proven that maintaining normal MLH1 expression levels is beneficial for maintaining hPMS1 (human post-meiotic segregation 1) and hPMS2 (human post-meiotic segregation 2) levels; that is to say, in the absence of MLH1, these proteins are unstable. Furthermore, the possible influence of smoking on MLH1 methylation has been suggested and it is understood that this could be a reversible process [17]. MGMT methylation is a well-described biomarker in several solid tumors, although on a clinical basis it is simply used in glioblastoma. MGMT is considered a good prognosis biomarker given that its own expression can be silenced, which results in cell death, particularly inducing cancer cells apoptosis [18].

The aims of this study were twofold: (i) to evaluate MHL1 and MGMT methylation levels in OSCC and OPMD to ascertain their prognostic and clinicopathological significance in our institutional database and (ii) to integrate this information with The Cancer Genome Atlas (TCGA) database.

## 2. Materials and Methods

### 2.1. Patients and Samples

For this study, eighty-five samples were selected from patients diagnosed with OSCC. The samples were divided as follows: (1) patients diagnosed with OSCC (OSCC group comprised of sixteen samples), (2) patients with OPDM who developed OSCC in the same location (transformation group comprised of 47 samples) and (3) patients with OPDM who developed OSCC in a different location (simultaneous group comprised of 22 samples). The samples were obtained from the Department of Oral Medicine of the University of Santiago de Compostela.

Patients included in this study were required to sign an informed consent form, and, subsequently, all of the samples were processed at the Pathological Anatomy Service of the University Hospital Complex of Santiago de Compostela (CHUS, by its acronym in Spanish). This project was approved by the Galician Clinical Research Ethics Committee (Register Code: 2019/271). For this study, the principles of the Declaration of Helsinki and its subsequent amendments were followed. The STROBE guidelines were used as the reference for data reporting [19]. 

### 2.2. Study of Methylation by MLH1 and MGMT Pyrosequencing

In order to determine the percentage of methylation of the samples studied in the MLH1 and MGMT genes, pyrosequencing analysis was conducted. Samples were initially embedded in paraffin. The total DNA was extracted and treated with bisulphite, using the Epitect Fast DNA Bisulfite Kit (Qiagen^®^, Hilden, Germany), and following the protocol recommended by the manufacturer: one 5 μm cut of paraffin for MHL1 and two 5 μm cuts for MGMT. The concentration of the samples was measured using the NanoDrop 2000 (Thermo Fisher Scientific, Waltham, MA, USA), and all of the samples had 260/280 values of between 1.7–2. Once transformed, the DNA was extracted, and the Therascreen MGMT Pyro kit (Qiagen^®^) was then used to perform PCR and pyrosequencing for methylated MGMT detection. With regards to MLH1, the PCR was performed using the Pyromark PCR kit 200 (Qiagen^®^) with a concentration of no greater than 100 ng/microliter. The pyrosequencing was conducted using Pyromark Q24 CPG MLH1 (4 × 24) (Qiagen^®^). All PCRs were performed in the Agilent Technologies Surecycler 8800, and the pyrosequencing was performed by the Pyromark Q24 and Pyromark Q24 workstation (Qiagen^®^), using the Pyromark Q24 2.0.7 to compute and analyze the results.

### 2.3. Methylation Quantification

For each sample, the average of the four and five CpG islands were calculated respectively, and the result was considered positive (meaning methylated) if the value was between 11 and 15% (as indicated by Qiagen^®^). In any cases in which the result was uncertain, the corresponding CpG island and Pyrogram were checked separately.

### 2.4. Variables and Collected Data

The qualitative variables determined were as follows: study group (OSCC, transformation and simultaneous), gender (male or female), area (gum, tongue, buccal mucosa, hard palate, floor of the mouth, retromolar trigone, labial mucosa, soft palate or tonsil), initial disorder and subsequent progression (leukoplakia without dysplasia, low degree leukoplakia, high degree leukoplakia, oral lichen planus, papilloma, verrucous carcinoma, non-specific ulcer or OSCC), exitus and qualitative MLH1 and MGMT methylation (yes > 11%, no < 11% or poorly 11–15%).

### 2.5. TCGA Cohort Sample Selection and Data Downloading

As a validation cohort in this study, data from The Cancer Genomic Atlas (TCGA) database, particularly regarding Head and Neck Squamous Cell Carcinoma (HNCS) was used [20]. The query was restricted to primary OSCCs. Data of the samples were retrieved from GDAC Broad Institute (https://gdac.broadinstitute.org/, accessed on 15 March 2022). After this collection, data was catalogued and manually checked based on the primary site of tumour onset in order to exclude non-OSCC patients, as aforementioned. After the filtration, 342 cases were left and downloaded directly from the server and entered into the cBioPortal for Cancer Genomics (http://www.cbioportal.org, accessed on 17 March 2022). Data for TCGA methylation derived from the Human Methylation-450 Bead Chip assay of MGMT and MLH1 was then gathered. As described elsewhere, for genes with multiple CpG-Islands, as in the case of MGMT, only methylation data from the probe with the strongest negative correlation between the methylation signal and the gene expression were chosen for further analysis. In order to identify the dichotomized methylation status (i.e., methylated or not) for the TCGA cohort, k-means clustering was performed, using beta values for selected CpG sites as initially reported by the TCGA program [20]. 

### 2.6. Statistical Analysis

The categorical variables were reported as frequencies, while the percentages and quantitative variables were displayed using means and standard deviation. In order to study the relationship between the categorical variables, the chi-square test was used and contingency tables were built. Similarly, to describe the differences in the means, the Student’s T-test or one-way ANOVA analysis was used, depending on the data distributions. Survival plots were created based on Kaplan–Meier curves and their related log ranks. SPSS v.24.0 (IBM, Statistics, NY, USA) software for Windows was used to perform this analysis. 

All the raw data obtained from the TCGA database was entered in R software v.4.1.0 and its JMV package to perform statistical analyses of correlation with overall survival (in months) for both MGMT and MLH1 methylation among the cohort. Also, correlation analysis was performed to check the mutual exclusivity of the methylation of both genes. For the sake of exhaustivity, both types of correlation estimates were presented (i.e., Spearman and Pearson correlation test), although the most appropriate figure was considered Spearman’s coefficient based on database constructors. Multiple linear regression models were conducted to more formally assess the results. Lastly, bearing in mind the post hoc analysis of our institutional cohort, we opted to perform a bioinformatic analysis to ascertain the pathways of the neoplasm involved in the methylation of MGMT and MLH1. Figure plotting was performed using the in-app service implemented in cBioPortal. The level of significance established for the results was *p* ≤ 0.05.

## 3. Results

### 3.1. Sample Description

The sample was comprised of 16 (18.8%) OSCCs, 47 (55.3%) previous OPDMs transformed lesions and 22 (25.9%) lesions in patients with simultaneous neoplasia with OPDMs in another location in the oral topography. In addition, 48 (56.5%) of the samples were from women and the tongue was the most common location (31.8%), followed by the buccal mucosa (28.2%) and the gingiva (15.3%). With regards to age, the average age was 70 ± 10.8 years old. The full descriptive data is shown in Table 1.

### 3.2. Methylation State

Overall methylation levels of 8.6 ± 11.5% for MHL1 and 8.1 ± 9.2% for MGMT were obtained. If assessed in a dichotomous way, 25.3% of the samples were methylated for MHL1 (1.2% poorly methylated) and 26.7% were methylated for MGMT (2.4% poorly methylated). Table 2 shows the methylation levels in the different study groups. With regards to MHL1, the OSCC presented the highest degree of methylation with 9.3 ± 7.3% (95%CI 5.1–13.6), and with regards to MGMT, the simultaneous malignancy group presented the highest degree of methylation with 10 ± 13.5% (95%CI 6–10). No significant differences were found between the groups (*p* = 0.934 and *p* = 0.515, respectively) (Table 3). No differences were found in terms of gender (*p* = 0.120 for MLH1 and *p* = 0.444 for MGMT), area (*p* = 0.780 for MLH1 and *p* = 0.506 for MGMT), recurrence (*p* = 0.358 for MLH1 and *p* = 0.561 for MGMT) or survival in the follow-up period (*p* = 0.680 for MLH1 and *p* = 0.413 for MGMT).

### 3.3. Follow-Up

The mean follow-up time was 60.5 ± 43.7 months, with a timelapse until malignant transformation of 33.4 ± 39.6 months, in which time 17 patients (20%) passed away, and 31 (36.5%) experienced recurrence. In terms of overall survival, it was 9.2 ± 6.5 months, whilst disease-free survival was 29 ± 26.7 months. The estimated survival in accordance with the Kaplan–Meier curves was higher for MGMT methylated cases (19.1 months, 95%CI 19.1–19.1) than for unmethylated cases (9.4 months, 95%CI 6–12.8) (Table 4). However, no statistically significant differences were observed (log rank, *p* = 0.134). With regards to the estimated time until recurrence, this was longer in MHL1 methylated cases (42.4 months, CI 95% 9.9–74.9) than in unmethylated cases (19.7 months, 95%CI 12–27.3), and no significant differences were found (log rank, *p* = 0.137).

### 3.4. Validation in External Dataset

**Figure 1:**
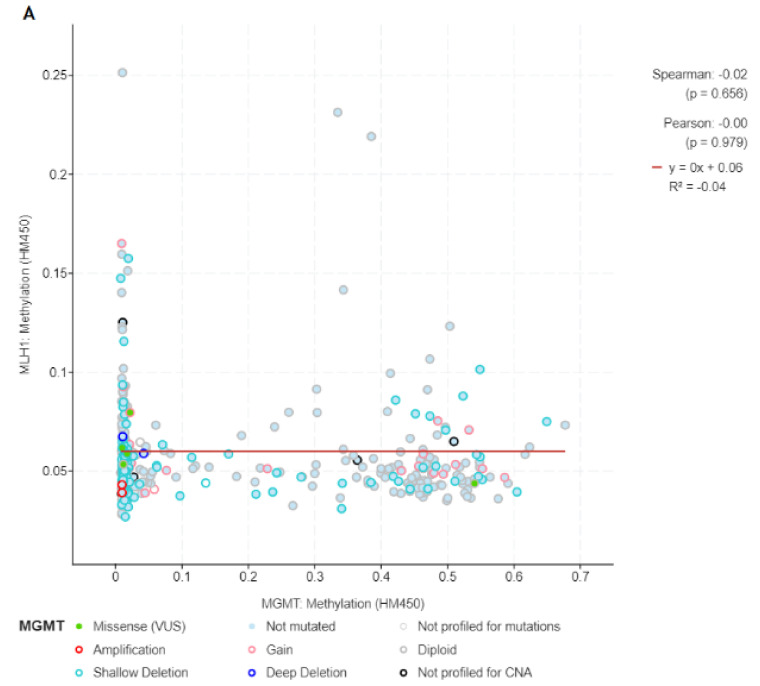

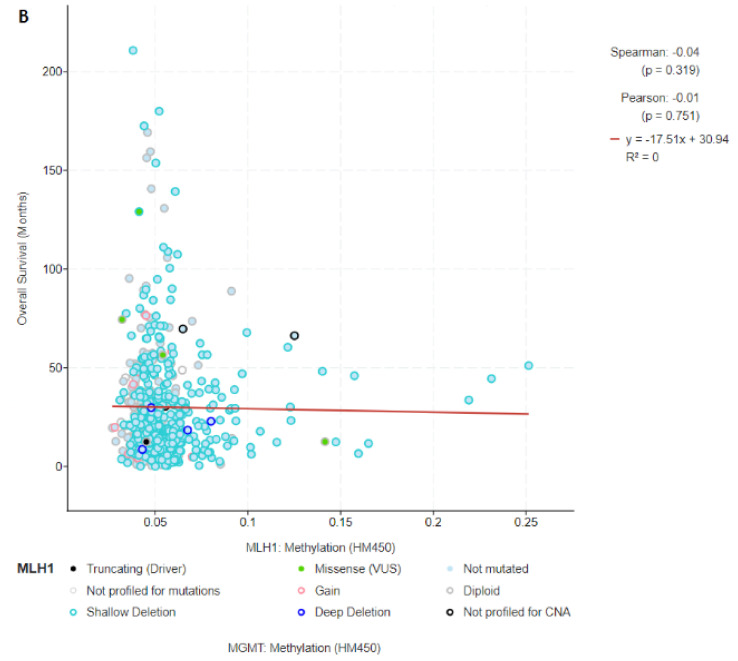

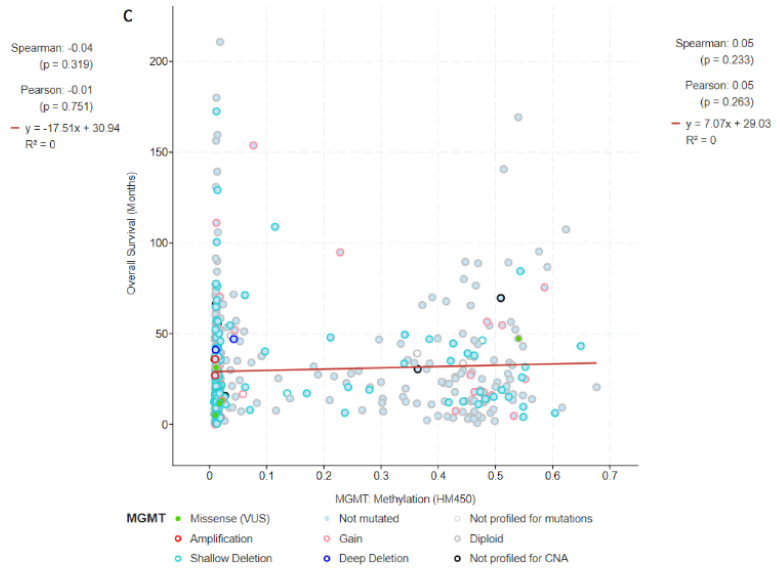
Integrative analysis with the TCGA database. (**A**) Correlation and linear regression model of MGMT and MLH1 methylation. Correlation and linear of MLH1 (**B**) and MGMT methylation (**C**) with overall survival in months.

Although it was not the intention, restrictions in order to exclusively include OSCC cases were discarded due to the poor levels of methylation in targeted genes within the cohort. In this vein, all head and neck squamous cell carcinomas (HNSC) that provided data on the matter under study were included (n = 504). The k- means clustering analysis clearly separated four groups of samples. According to the analysis of available TCGA data, 495 tumour samples were hypomethylated, while nine tumour samples were hypermethylated for MGMT. In the case of MLH1, the figures were quite similar with only nine cases considered as hypermethylated according to the Methylation-450 Bead Chip assay. Assuming that the correlations of MGMT and MLH1 methylation are A and B, respectively, we performed a pooled analysis of Log2 Odds Ratio to study mutual exclusivity. Log2 Odds Ratio was <−3 showing a q-value of 0.849 derived from the Benjamin–Hochberg false discovery ratio correction procedure (i.e., no patient shows a double methylation for both genes). This asseveration is represented by the non-significant levels of correlation and regression analysis as displayed in Figure 1A. 

Owing to the poor levels of hypermethylation, the intention to perform a Kaplan–Meier analysis was discarded and a correlation analysis of MGMT and MLH-1 versus overall survival analysis (in months) was computerised. The analysis is displayed in Figure 1C in the case of MGMT and Figure 1B for MLH1. MGMT hypermethylation was not correlated with overall survival (Spearman’s correlation coefficient = −0.05, *p* = 0.233). Similarly, hypermethylation of MLH-1 was also not correlated with overall survival (Spearman’s correlation coefficient = −0.04, *p* = 0.319). Figure 1 displays the mathematical analysis by means of regression models. 

**Figure 2:**
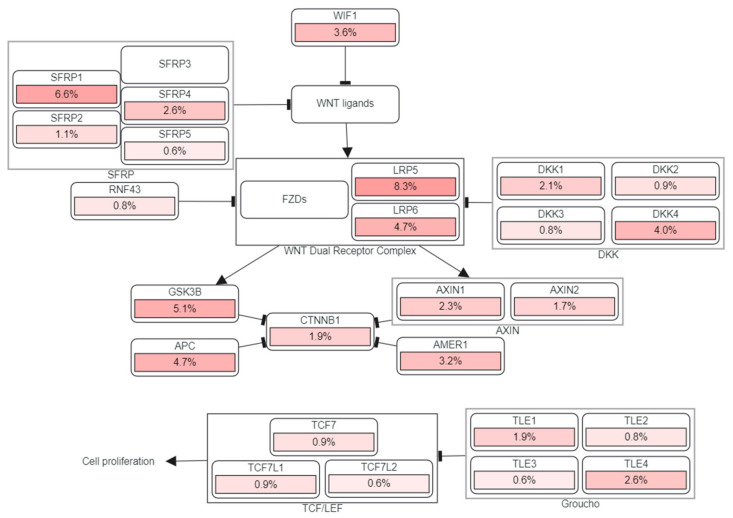
Pathways involved in head and neck carcinogenesis related to MLH1 and MGMT methylation according to the TCGA database.

Lastly, taking the methylation of both genes together we indagated the pathways involved in carcinogenesis in the portion of the TCGA database under study. The most affected pathway seemed to be WNT. These genes can act upon WNT ligands, affecting subsystems such as those involving genes which play pivotal roles in the WNT dual receptor complex, SFRP, DKK and AXIN. At the same time, this orchestrated state can induce cell proliferation through a molecular machinery involving the Groucho/TLE/Grg family of transcriptional co-repressors and TCF/LEF Transcription Factors (Figure 2).

## 4. Discussion

Despite epigenetic alterations found in OSCC and OPMD methylation levels and their correlations with clinical outcomes and survival in the present study, our results are inconsistent with the previous literature [21]. These differences reflect the heterogeneity of these diseases in their histology and clinical behavior, with different aetiologias and risk factors, and known tissue and tumor-type specificity of methylation patterns [22]. The prevalence of MGMT and MLH1 methylation detected in this study are also consistent with previously summarized data for HNSC in the TCGA database [23,24]. 

The determination of MGMT promoter methylation is of great interest to clinicians and patients, given that in recent in vitro studies with cell lines, an association between MGMT promoter methylation and OSCC diagnosis has been observed compared to normal cell lines. Furthermore, this repression was reversed following treatment with a demethylating agent (5-Aza-2′-deoxycytidine) [25], which was corroborated by an increase in gene expression [23]. However, the methylation percentage obtained in our samples was not significant for any clinicopathological or survival feature. 

The percentage of methylated OSCC cases with MGMT was 9.2%, which was less than the percentage obtained in other studies with a similar sample size. In those studies, a methylation percentage of 27% was obtained, and in their control group, a methylation percentage of 7% was obtained [26]. This team has also observed that combined methylation of p16 and MGMT promoters was only found in the OSCC group. In this study, combined methylation between MGMT and MHL1 genes was analyzed and no association with specific clinical parameters, such as survival or recurrence, was found. Recent meta-analyses have also suggested that MGMT methylation may be related to OSCCs which metastasise; however, these results did not reveal a worse prognosis in methylated samples [27]. A study conducted by Taioli et al. observed the association between MGMT methylation and a poorer prognosis for OSCC [28]. However, our analyses found a higher survival rate in MGMT-methylated cases (19.1 months, 95%CI 19.1–19.1) than in unmethylated cases (9.4 months, 95%CI 6–12.8) without any statistically significant difference. On the other hand, in the present study, MGMT methylation was more marked in the simultaneous malignization group, obtaining 10 ± 13.5%. Based on the field cancerization theory, the DNA methylation results may reflect that the entire epithelial histology adjacent to tumors and premalignant lesions have prominent levels of methylation of this gene, suggesting that MGMT methylation is an early event in oral carcinogenesis [10]. Nonetheless, the methylation of other genes in mouth neoplasm such as APC, DAPK, ECAD, RASSF1, TIMP3 and p16, have retrieved more promising diagnostic and prognostic values [21]. 

Fewer studies have considered the methylation of the MLH1 gene in oral cancer compared with the MGMT [29,30]. A study found that 100% of mouth neoplasm is affected by this gene methylation, which suggests that this occurs in patients with an advanced stage of the disease [16]. This does not coincide at all with the results obtained in this study, nor with those obtained by Ramirez et al., who suggested that it is an early marker for malignancy in oral lesions [15]. However, according to our results, both OPDMs groups in our institutional cohort did not verify these results, nor did our integration with the TCGA database. 

The strength of our study is the well-characterized population of the groups OPMD and OSCC, which has detailed baseline epidemiological data on risk factors and also a comprehensive follow-up. An obvious limitation of the present study is its cross-sectional design and the small sample size. On the other hand, one of the main aims of this study was to assess early methylation in both genes in oral lesions prior to malignancy and particularly its implication in field cancerization. However, methylation in the OPDM groups was not significant, nor was it associated with the correspondent OSCC samples with methylation patterns maintained over time. In this sense, the present results preclude the generalization of results.

## 5. Conclusions

In conclusion, our results did not show a correlation between MGMT and MLH1 gene methylation and any clinicopathological feature or survival in our institutional cohort. Moreover, our results showed that MLH1 methylation was present mainly in OSCC, whilst MGMT in OPMD represented a modest contribution to field cancerization. There was an overall consistency with the TCGA database, although it should be borne in mind that individually the methylation of these genes has not proven clinical significance. Based on the results of this study, we encourage the development of further studies analyzing the methylation of more genes prospectively in bigger cohorts to unravel a panel able to reach clinical translation.

## Figures and Tables

**Table 1:** Characteristics of the institutional cohort and levels of methylation.

Qualitative Variables			N		%
Study group	OSCC		16		18.8
	Transformation		47		55.3
	Simultaneous		22		25.9
Gender	Woman		48		56.5
	Man		37		43.5
Location	Gingiva		13		15.3
	Tongue		27		31.8
	Buccal mucosa		24		28.2
	Hard palate		2		2.4
	Floor mouth		2		2.4
	Retromolar trigone		8		9.4
	Lip		3		3.5
	Soft palate		3		3,5
	Tonsil		3		3.5
Exitus	No		68		80.0
	Yes		17		20.0
Relapse	No		54		63.5
	yes		31		36.5
Treatment	Only surgery		56		65.9
	Radiotherapy		22		25.9
	Radiotherapy and chemotherapy		7		8.2
Quantitative variables	N	Minimum	Maximum	Average	SD
Percentage MLH1	75	0.4	63.8	8.6	11.5
Percentage MGMT	82	1.0	41.0	8.0	9.2
Time until malignancy	58	0.4	116.6	33.4	39.4

**Table 2:** Methylation levels stratified according to subgroups of our institutional database.

Lesion		Study Group	MLH1 Methylation		*p*-Value	MGMT Methylation		*p*-Value
			No	Yes		No	Yes	
Initial lesion	Leukoplakia without dysplasia	Transformation	10 (55.6%)	3 (75.0%)	0.474	11 (64.7%)	3 (60.0%)	0.848
		Simultaneous	8 (44.4%)	1 (25.0%)		6 (35.3%)	2 (40.0%)	
	Oral lichen planus	Transformation	2 (40.0%)	0 (0.0%)	0.439	2 (50.0%)	0 (0.0%)	0.221
		Simultaneous	3 (60.0%)	1 (100.0%)		2 (50.0%)	2 (100.0%)	
	Papilloma	Transformation	2 (100.0%)			2 (100.0%)		
		Simultaneous						
	Verrucous carcinoma	Transformation		1 (100.0%)		1 (100.0%)		
		Simultaneous						
	Erythroplasia	Transformation				1 (100.0%)		
		Simultaneous						
	Nonspecific ulcer	Transformation	2 (100.0%)	2 (100.0%)		2 (100.0%)	2 (100.0%)	
		Simultaneous						
Final lesion	Leukoplakia without dysplasia	Transformation	2 (50.0%)	1 (100.0%)	0.361	3 (60.0%)	1 (100.0%)	0.439
		Simultaneous	2 (50.0%)	0 (0.0%)		2 (40.0%)	0 (0.0%)	
	Leukoplakia low grade	Transformation	6 (85.7%)	1 (50.0%)	0.284	6 (60.0%)	1 (100.0%)	0.428
		Simultaneous	1 (14.3%)	1 (50.0%)		4 (40.0%)	0 (0.0%)	
	Leukoplakia high grade	Transformation	2 (100.0%)	1 (100.0%)		5 (100.00%)		
		Simultaneous						
	Oral lichen planus	Transformation	1 (33.3%)	0 (0.0%)	0.505	1 (50.0%)		
		Simultaneous	2 (66.7%)	1 (100.0%)		1 (50.0%)		
	Papilloma	Transformation	2 (100.0%)			1 (100.0%)	1 (100.0%)	
		Simultaneous						
	Verrucous carcinoma	Transformation	3 (100.0%)			3 (100.0%)		
		Simultaneous						
	Nonspecific ulcer	Transformation	2 (100.0%)	1 (100.0%)		2 (100.0%)	2 (100.0%)	
		Simultaneous						
	OSCC	OSCC	8 (25.0%)	6 (42.9%)		12 (33.3%)	4 (30.8%)	0.825
		Transformation	13 (40.6%)	6 (42.9%)		16 (44.4%)	5 (38.5%)	
		Simultaneous	11 (34.4%)	2 (14.3%)		8 (22.2%)	4 (30.8%)	

**Table 3:** Clinicopathological features relationship with MLH1 and MGMT methylation.

Methylation			Average	Standard Deviation	Confidential Interval 95%		*p*-Value
					Lower Limit	Upper Limit	
MLH1 methylation	Study group	OSCC	9.3	7.3	5.1	13.6	0.934
		Transformation	8.7	13.8	4.3	13.0	
		Simultaneous	7.9	8.8	3.8	11.9	
	Sex	Woman	6.8	9.3	3.9	9.7	0.120
		Man	11.0	13.8	5.9	16.1	
	Location	Gingiva	10.6	18.0	−1.5	22.7	0.780
		Tongue	11.5	13.4	5.9	17.0	
		Buccal mucosa	6.9	8.2	3.0	10.7	
		Hard palate	2.4	−	−	−	
		Floor mouth	3.2	0	3.2	3.2	
		Retromolar trigone	4.8	3.9	1.2	8.4	
		Lip	3.5	1.3	0.3	6.7	
		Soft palate	11.3	10.0	−13.6	36.3	
		Tonsil	4.8	4.7	−6.8	16.4	
MGMT methylation	Study group	OSCC	6.6	6.4	3.2	10.0	0.515
		Transformation	7.7	7.7	5.4	10.0	
		Simultaneous	10.0	13.5	3.5	16.5	
	Sex	Woman	7.3	9.1	4.6	10.0	0.444
		Man	8.9	9.4	5.7	12.1	
	Location	Gum	9.4	11.4	2.1	16.6	0.506
		Tongue	5.8	5.9	3.5	8.2	
		Buccal mucosa	10.9	12.0	5.6	16.2	
		Hard palate	8.5	6.4	-48.7	65.7	
		Floor mouth	2.1	0.5	−2.6	6.9	
		Retromolar trigone	5.1	5.1	0.8	9.4	
		Lip	4.3	1.9	−0.3	8.9	
		Soft palate	12	10.3	−13.5	37.5	
		Tonsil	12.8	13.9	−21.9	47.4	

**Table 4:** Association of MLH1 and MGMT methylation with survival and recurrences among the cohort.

Kaplan Meier			Months (Mean)	Confidential Interval 95%		Log Rank Test *p* Value
				Lower Limit	Upper Limit	
Survival	MLH1 methylation	No	9.1	5.2	13.1	0.827
		Yes	9.3	1.3	17.3	
	MGMT methylation	No	9.4	6.0	12.8	0.134
		Yes	19.1	19.1	19.1	
Recurrence	MLH1 methylation	No	19.7	12.0	27.3	0.137
		Yes	42.4	9.9	74.9	
	MGMT methylation	No	30.9	19.2	42.6	0.267
		Yes	18.2	9.4	26.9	

## Data Availability

Not applicable.
